# Ghrelin and adipokines as circulating markers of disease activity in patients with Takayasu arteritis

**DOI:** 10.1186/ar4120

**Published:** 2012-12-21

**Authors:** Hatice Yilmaz, Vedat Gerdan, Didem Kozaci, Dilek Solmaz, Servet Akar, Gercek Can, Aytac Gulcu, Yigit Goktay, Ismail Sari, Merih Birlik, Nurullah Akkoc, Fatos Onen

**Affiliations:** 1 Department of Internal Medicine, Dokuz Eylul University School of Medicine, Inciralti/Izmir, 35340, Turkey; 2 Department of Internal Medicine, Division of Rheumatology, Dokuz Eylul University School of Medicine, Inciralti/Izmir, 35340, Turkey; 3 Adnan Menderes University Science and Technology Research Center, Aydin, 09010, Turkey; 4 Department of Radiodiagnostics, Dokuz Eylul University School of Medicine, Inciralti/Izmir, 35340, Turkey

## Abstract

**Introduction:**

The current markers of disease activity in Takayasu arteritis (TA) are insufficient for proper assessment. We investigated circulating levels of unacylated and acylated ghrelin, leptin and adiponectin and their relationships with disease activity in patients with TA.

**Methods:**

This study included 31 patients with TA and 32 sex-, age- and body mass index-matched healthy controls. Disease activity was assessed in TA patients using various tools, including Kerr's criteria, disease extent index-Takayasu, physician's global assessment, radiological parameters, and laboratory markers. Plasma unacylated and acylated ghrelin, and serum leptin and adiponectin levels were measured using an enzyme-linked immunosorbent assay.

**Results:**

Unacylated and acylated ghrelin levels were found to be significantly lower in TA patients than that in healthy controls. Patients with active disease had lower unacylated ghrelin levels than those with inactive disease and had lower acylated ghrelin levels than healthy controls. Ghrelin levels were negatively correlated with various parameters of disease activity. The leptin/ghrelin ratio was significantly higher in TA patients than controls. It was positively correlated with disease activity. There was a positive correlation between unacylated and acylated ghrelin and a negative correlation between leptin and ghrelin. There was no statistical difference in adiponectin levels between TA patients and controls. The radiological activity markers were positively correlated with other parameters of disease activity.

**Conclusions:**

This study suggests that plasma unacylated and acylated ghrelin levels may be useful in monitoring disease activity and planning treatment strategies for patients with TA. The serum leptin level and leptin/ghrelin ratio may also be used to help assess the disease activity.

## Introduction

Takayasu arteritis (TA) is a granulomatous panarteritis affecting large vessels, predominantly the aorta and its main branches. The disease is characterised by an acute phase with constitutional symptoms and followed by a vascular phase presented with symptoms due to stenosis, occlusion or aneurysms. However, these phases often overlap during the disease course [1].

The diagnosis of TA is based on its suggestive clinical features and imaging methods such as conventional angiography, magnetic resonance angiography (MRA) and ultrasonography (US). Conventional angiography is the gold standard for diagnosis. It shows clear outlines of the lumen of affected arteries, whereas MRA and US can demonstrate thickening of the vascular wall of the aorta and its major branches [2-5].

Monitoring the disease activity is of great importance because intensive treatment is required in the active periods of TA. However, until now a reliable serological marker of disease activity has not been identified. Acute-phase reactants, such as erythrocyte sedimentation rate (ESR) and serum C-reactive protein (CRP) may demonstrate active inflammation but their specificity and sensitivity are quite low [6]. ESR is increased in nearly half of the patients in clinical remission, while it is normal in 28% of the patients with active disease [7]. The current criteria for the assessment of disease activity in TA [7] are not sufficient either.

Ghrelin, a recently discovered hormone, is produced primarily by cells in the stomach but is also expressed in white adipose tissue [8]. It has been shown to affect a number of different systems including growth hormone release, feeding, gastric acid secretion, gastric motility, and cell proliferation. Ghrelin and its receptor have been identified in T cells. It is a potent anti-inflammatory mediator in lymphocytes, monocytes and dendritic cells. It inhibits oxidative stress, cellular apoptosis, cell adhesion and proinflammatory cytokine expression and promotes IL-10 expression and cell migration [9].

Ghrelin is expressed in two different forms, namely acylated ghrelin and unacylated ghrelin. Initially, the unacylated ghrelin was considered to be biologically inactive but then a role of unacylated ghrelin in controlling the growth hormone (GH)-insulin-like growth factor 1 (IGF-I) axis has been demonstrated. Moreover, both acylated and unacylated forms of ghrelin have been shown to bind to common sites on cardiomyocytes and endothelial cells mediating similar signals to inhibit cellular apoptosis [10].

Adipokines, leptin and adiponectin are polypeptid hormones produced mainly by adipose tissue. Leptin regulates food intake and energy balance. It is now regarded as a pivotal factor in the interplay between neuroendocrine function and the immune system [11,12]. Proinflammatory cytokines increase circulating leptin, which in turn triggers cytokine release in monocytes/macrophages and stimulates T cell-mediated immunity during acute inflammation [13,14]. However, leptin may also limit the inflammatory response [15,16]. Adiponectin has antidiabetic and anti-atherogenic effects. It also appears to have anti-inflammatory properties because of its antagonism against TNF-α [17].

To our knowledge, there is no study investigating ghrelin and adipokines in TA, an inflammatory disease that is frequently accompanied by aneroxia and weight loss. In the current study, we aimed to investigate circulating unacylated and acylated ghrelin, leptin and adiponectin levels and to evaluate their relationships with disease activity in patients with TA.

## Materials and methods

### Patients and controls

Patients with TA followed up in the Division of Rheumatology, Dokuz Eylul University Medical Faculty (Izmir, Turkey) were called by telephone and invited to the hospital to participate in this study. We obtained detailed medical history and performed full physical examination on each patient who agreed to take part in this study. Thirty-two sex-, age- and body mass index (BMI)-matched hospital workers were evaluated as healthy controls.

The study was approved by the Dokuz Eylul University Ethics Committee for Non-invasive Clinical Research (project ethics number: B.30.2.DEU.0.01.00.00/15482). Written informed consent was obtained from all participants before enrollment.

### Assessment of disease activity

Disease activity was assessed using various tools including Kerr's criteria [7], disease extent index-Takayasu (DEI.Tak) and physician's global assessment (PGA) [18], radiological parameters and acute-phase reactants.

Kerr's criteria [7] are used to define active disease if two of the following criteria are positive: 1) systemic features with no other cause; 2) elevated ESR; 3) features of vascular ischemia or inflammation (claudication, diminished or absent pulses, bruit, vascular pain, asymmetric blood pressure; or 4) typical angiographic features (in this study, MRA was also included to evaluate new vessel involvement or disease progression).

DEI.Tak [18] is a new index that was developed for follow up examinations of TA patients. DEI.Tak assesses clinical findings (without imaging) at intervals of at least 6 months, but only includes new or worsening symptoms that have developed within the past 6 months. TA patients with a DEI.Tak score ≥ 1.0 at the time of this study were considered to have active disease. PGA is an item included on DEI.Tak, in which the physician classifies the disease status according to one of three categories: active disease, grumbling/persistent disease, or inactive disease.

Digital subtraction angiography (DSA) had been performed on all except one of the TA patients by the time of diagnosis. According to the TA follow up protocol of the Rheumatology and Radiology Board of our hospital, the TA patients are followed using three-monthly B-mode/ Doppler US and annual magnetic resonance imaging (MRI) with MRA examinations. Bilateral carotid, upper and lower extremity, renal and mesenteric arterial and abdominal aorta Doppler US examinations were performed to evaluate flow parameters and vessel wall thickness in all TA patients. MRI examinations, including examination of the aortic arch and supra-aortic arteries with specific investigations of the vessel walls and black-blood images were obtained before and after injection of a paramagnetic contrast agent to evaluate vessel wall enhancement. MRA examinations were performed to evaluate the whole aorta and its main branches.

We categorized the patients as having active disease based on the results of the radiological examinations if any of the following findings were positive: 1) new vessel involvement, as determined by any of the radiological studies including DSA, MRA and Doppler US; 2) increase in the carotid intima-media thickness (IMT) compared with a previous US examination; 3) the presence of vessel wall thickening and contrast enhancement on MRI.

All US and Doppler US examinations were performed by the same radiologist, as previously described [19], using an HDI-5000scanner (Advanced Technology Laboratories, Bothell, Washington, DC, USA) equipped with 12 to 5 MHz and 7 to 5 MHz linear array imaging probes. MR studies were performed by an Achieva 1.5T MRI scanner (Philips Healthcare, Best, The Netherlands).

The serum C-reactive protein level (CRP, normal range 0.1 to 8.2 mg/L), erythrocyte sedimentation rate (ESR, normal range 0 to 20 mm/hour), and total leukocyte and neutrophil numbers were measured as laboratory parameters of disease activity in TA patients and compared with those in the controls.

### Measurement of unacylated and acylated ghrelin, leptin, and adiponectin

Overnight fasting-state blood samples (K2 ethylenediaminetetraacetic acid (EDTA)-treated and not) were obtained from patients and controls. Plasma leptin, adiponectin, unacylated ghrelin and acylated ghrelin levels were measured by ELISA kits, commercially available from Biovendor RDP (Brno, Czech Republic). The assays were performed according to the manufacturer's instructions. ELISA plates were coated with anti-human leptin, adiponectin, unacylated ghrelin and acylated ghrelin antibodies, respectively. Color was developed with 3,3',5,5'-tetramethylbenzidine (TMB) and absorbance was measured at 450 nm. Mean values were reported.

### Statistics

The Kolmogorov-Smirnov test was used to determine if each parameter followed a normal distribution. The values are expressed as the mean ± SD, median, or percentage, as appropriate. For comparison of the groups, Kruskal-Wallis analysis or chi-square tests were used. When between-group significance was determined, data were compared in pairs using the Mann-Whitney *U*-test. Relationships were determined using the phi coefficient, Spearman's rho, or intraclass correlations. All statistical analyses were performed using SPSS software (version 15.0; SPSS Inc., Chicago, IL, USA). Differences were considered significant at *P *< 0.05.

## Results

### Demographic and clinical features

In total, 31 patients (28 female and 3 male) who fulfilled the American College of Rheumatology (ACR) criteria for TA [20] were enrolled in this study. Their mean age was 44.2 years, and the median disease duration was 7 years (range 1 to 30 years). Thirty-two healthy sex-, age- and BMI-matched hospital workers (28 female and 4 male, mean age 41.5 years) were evaluated as controls. The demographic, clinical and laboratory features of patients and controls are shown in Table 1.

**Table 1:** Characteristics of the patients with Takayasu arteritis and matched healthy controls

	Takayasu patients (*n *= 31)	Healthy controls (*n *= 32)	*P*
Age, years, mean ± SD	44.2 ± 11.3	41.5 ± 5.9	0.22
Sex, female/male, n	28/3	28/4	1.00
Body mass index, mean ± SD	25.2 ± 4.2	25.0 ± 3.3	0.87
Waist circumference, cm, mean ± SD	86.6 ± 13.0	83.4 ± 10.0	0.28
Hip circumference, cm, mean ± SD	102.9 ± 9.2	104 ± 7.5	0.59
Diabetes mellitus, n (%)	3 (9.7)	0	
Hypertension, n (%)	12 (38.7)	0	
Hyperlipidemia, n (%)	9 (29)	0	
Coronary artery disease, n (%)	3 (9.6)	0	
Ever smoked, n (%)	4 (12.9)	0	
Menopausal, n (%)	11 (39.3)	0	
White blood cells, n/uL, mean ± SD	8622 ± 3614	6681 ± 1237	0.002
Neutrophil, n/uL, mean ± SD	5754 ± 2747	3878 ± 1000	0.001
Hemoglobin, g/dL, mean ± SD	12.5 ± 1.7	12.9 ± 1.6	0.21
ESR, mm/hour, mean ± SD	28.3 ± 24.1	16.2 ± 8.5	0.017
C-reactive protein, mg/L, mean ± SD	11.7 ± 24.6	2.7 ± 3.0	0.11

ESR, erythrocyte sedimentation rate

According to the angiographic classification [21], eighteen patients with TA (58.1%) had type 5 vessel involvement, ten had type 1 (32.3%), two had type 4 (6.4%) and one had type 2 (3.2%). Coronary artery involvement was present in three of thirty-one TA patients. Nineteen TA patients (68.7%) were using corticosteroid treatment during the study period. Immunosuppressive treatments included the following: methotrexate monotherapy in thirteen patients (41.9%), methotrexate plus leflunomide in three patients (9.7%), azathioprine monotherapy in ten patients (32.3%), leflunomide monotherapy in three patients (9.7%), and azathioprine plus leflunomide in one patient (3.2%). One patient (3.2%) was not receiving treatment.

Five patients (16.1%) had undergone prior vascular surgery and twenty (64.5%) had undergone percutaneous angioplasty.

### Disease activity in TA patients

According to Kerr's criteria and the radiological definitions, six (20%) and ten (33%) TA patients respectively had active disease. Seventeen patients (53.1%) had a DEI.Tak score ≥ 1.0 at the time of the study; these patients were considered to have active disease. The mean DEI-Tak score was 1.0 at the time of the study. The increase in the mean DEI-Tak score value during the last six months was calculated as 0.17. When the patients were evaluated according to the criteria of PGA, we found that six (19.4%) patients had active disease, seven (22.6%) had grumbling/persistent disease and eighteen (58.1%) had inactive disease.

There was a significant positive correlation between radiological activity markers and Kerr's criteria, DEI-Tak and PGA (*r *= 0.408, *P *= 0.023; *r *= 0.463, *P *= 0.009; and *r *= 0.409, *P *= 0.031, respectively). Significant correlation was also determined between Kerr's criteria and DEI.Tak and Kerr's criteria and PGA (*r *= 0.566, *P *= 0.001 and *r *= 0.603, *P *= 0.001 respectively).

ESR and total leukocyte and neutrophil numbers were higher in TA patients than in controls (Table 1). We determined that the immunosuppressive treatment was changed in eight TA patients and the glucocorticoid dose was increased in three TA patients due to treatment failure during the last month. Both the change in immunosuppressive treatment and increase in glucocorticoid dose correlated well with Kerr's criteria (*r *= 0.640, *P *< 0.001 and *r *= 0.667, *P *< 0.001, respectively), PGA (*r *= 0.570, *P *= 0.002 and *r *= 0.400, *P *= 0.035, respectively) and the radiological activity markers (*r *= 0.370, *P *= 0.042 and *r *= 0.471, *P *= 0.009, respectively).

### Unacylated and acylated ghrelin levels

**Figure 1:**
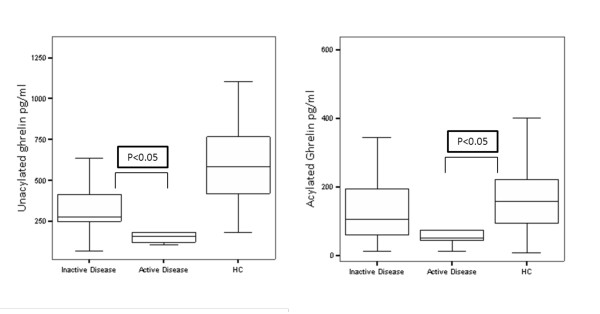
**Plasma ghrelin levels in patients with Takayasu's arteritis (TA) and matched healthy controls**. Patients with active disease had lower unacylated ghrelin levels than those with inactive disease (**A**) and had lower acylated ghrelin levels than healthy controls (**B**).

Plasma unacylated and acylated ghrelin levels (319.3 ± 202.6 pg/mL and 120.5 ± 94.4 pg/mL, respectively) were found to be significantly lower in TA patients than healthy controls (623.2 ± 270.0 pg/mL and 180.9 ± 128.7 pg/mL, respectively) (*P *< 0.001 and *P *< 0.05, respectively) (Table 2). Their levels were also lower in the patients with active disease than those with inactive disease according to PGA (*P *< 0.05 for unacylated ghrelin (Figure 1) although the difference in acylated ghrelin levels did not reach statistical significance. Furthermore, the level of acylated ghrelin was lower in TA patients with active disease than in healthy controls according to both PGA (*P *< 0.05) (Figure 1) and DEI-Tak (*P *< 0.05). There was no difference in acylated ghrelin levels between inactive patients and healthy controls (Figure 1).

**Table 2:** The levels of unacylated and acylated ghrelin, leptin, and adiponectin in patients and controls

	TA	HC	*P*
Unacylated ghrelin, pg/mL	319.3 ± 202.6	623.2 ± 270.0	< 0.001
Acylated ghrelin, pg/mL	120.5 ± 94.4	180.9 ± 128.7	< 0.05
Leptin, ng/mL	72 ± 62	52 ± 35	➢ 0.05
Adiponectin, µg/mL	16 ± 9	13 ±5	➢ 0.05

Results are presented as mean ± SD. TA, Takayasu arteritis; HC, healthy controls.

**Figure 2:**
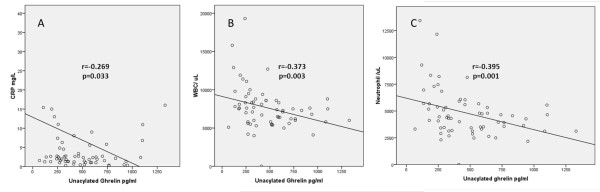
**Negative correlations between plasma unacylated ghrelin levels and serum C-reactive protein (CRP) (A) and white blood cell count (WBC) numbers (B) and neutrophil numbers (C)**.

**Figure 3:**
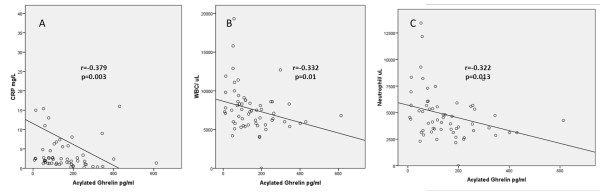
**Negative correlations between plasma acylated ghrelin levels and serum C-reactive protein (CRP) (A) and white blood cell (WBC) numbers (B) and neutrophil numbers (C)**.

The level of unacylated ghrelin was negatively correlated with DEI.Tak and PGA (r = -0.428, p = 0.037 and r = -0.501, p <0.001 respectively). There was also a negative correlation between unacylated ghrelin and CRP, and the numbers of leukocyte and neutrophil (r = -0.269, p = 0.033; r = -0.373, p = 0.003 and r = -0.395, p = 0.001, respectively) (Figure 2). The level of acylated ghrelin was also negatively correlated with CRP levels, and with leukocyte and neutrophil numbers (*r *= -0.379, *P *= 0.003, *r *= -0.332, *P *= 0.01 and *r *= -0.322, *P *= 0.013, respectively) (Figure 3).

**Figure 4:**
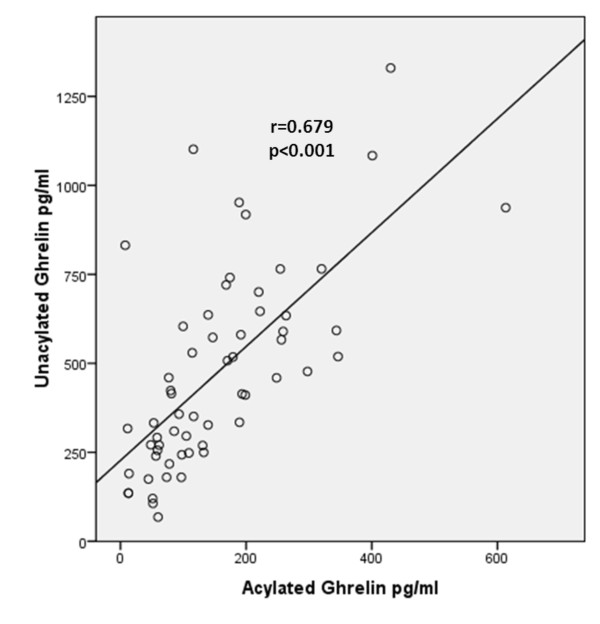
**Positive correlation between plasma unacylated and acylated ghrelin levels**.

The unacylated and acylated ghrelin levels were lower in patients who underwent changes in immunosuppressive medication than in patients who did not (*P *= 0.003 and *P *= 0.018, respectively). The unacylated and acylated ghrelin levels were also found to be lower in patients who underwent an increase in glucocorticoid dose than in patients who did not undergo an increase in glucocorticoid dose (*P *= 0.027 and *P *= 0.020, respectively). The levels of acylated and unacylated ghrelin in TA patients who had diabetes mellitus and hypertension were not statistically different from those who did not have these comorbidities (*P *> 0.05). Positive correlation was found between unacylated ghrelin and acylated ghrelin levels (*r *= 0.679, *P *< 0.001) (Figure 4).

### Leptin and adiponectin levels

**Figure 5:**
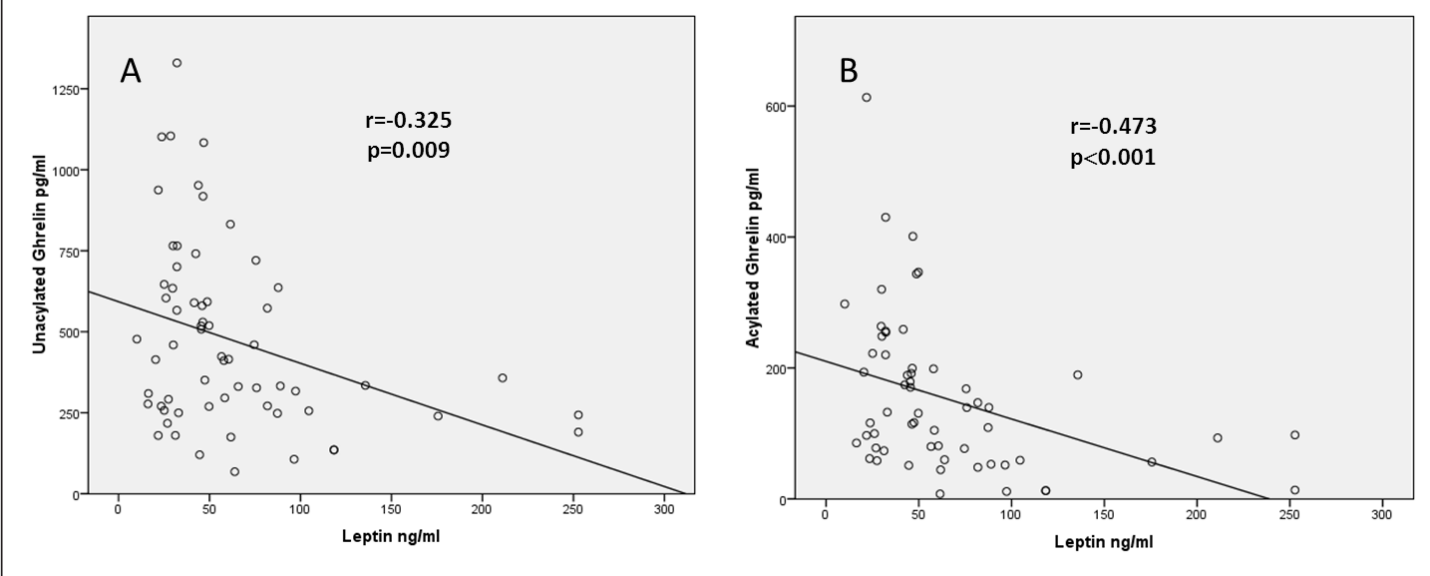
**Negative correlations between serum leptin and plasma unacylated ghrelin (A) and acylated ghrelin levels (B)**.

Leptin level was higher in TA patients than in healthy controls but the difference did not reach statistical significance (Table 2). It was positively correlated with neutrophil numbers (*r *= 0.262, *P *= 0.038). There was negative correlation between leptin and unacylated and acylated ghrelin levels (*r *= -0.325, *P *= 0.009 and *r *= -0.473, *P *< 0.001, respectively) (Figure 5). The level of leptin was higher in patients who underwent an increase in glucocorticoid dose than those who did not (*P *< 0.05).

There was no statistical difference in adiponectin levels between TA patients and healthy controls (Table 2). The level of adiponectin was not different in the patients with active disease compared with those with inactive disease and the controls.

### Leptin/ghrelin ratio

The ratio of leptin/ghrelin was significantly higher in TA patients than in healthy controls (*P *= 0.001). It did not differ between TA patients with active or inactive disease. The ratio of leptin/ghrelin was correlated with Kerr's criteria, DEI.Tak, PGA, the radiological activity parameters and neutrophil numbers (*r *= -0.367, *P *= 0.003; *r *= -0.370, *P *= 0.003; *r *= -0.332, *P *= 0.008; *r *= -0.377, *P *= 0.002; *r *= 0.356, *P *= 0.004, respectively).

### The effect of glucocorticoid treatment on ghrelin and leptin levels

No significant differences were found in unacylated and acylated ghrelin, and leptin levels between TA patients using glucocorticoid treatment and those not using glucocorticoid treatment (*P *= 0.162, *P *= 0.530 and *P *= 0.400, respectively).

### The relationships of ghrelin and adipokines with BMI, and waist and hip circumference

Unacylated and acylated ghrelin, and adiponectin levels were negatively correlated with BMI (*r *= -0.302, *P *= 0.016; *r *= -0.277, *P *= 0.033; and *r *= -0.306, *P *< 0.05, respectively), waist circumference (*r *= -0.423, *P *= 0.001; *r *= -0.445, *P *< 0.001 and *r *= -0.285, *P *= <0.05, respectively) and waist/hip ratio (*r *= -0.416, *P *= 0.001; *r *= -0.356, *P *= 0.006; *r *= -0.404, *P *= 0.001, respectively). A negative correlation between acylated ghrelin level and hip circumference (*r *= -0.353, *P *= 0.006) was also determined. The leptin level was positively correlated with BMI (*r *= 0.508, *P *< 0.001), waist circumference (*r *= 0.435, *P *< 0.001) and hip circumference (*r *= 0.546, *P *< 0.001).

### The carotid artery ultrasound results

The mean right and left carotid artery IMT measurements were 1.10 ± 0.61 mm and 0.92 ± 0.44 mm, respectively. Among 31 patients with TA, 14 had diffuse and homogeneous IMT >1 mm by B-mode US, which had been defined as suggestive for involvement with TA. Among these 14 patients, 10 (71.4%) were already known to have carotid artery involvement identified by angiography or MRA. There was no relationship between mean carotid IMT and leptin, or ghrelin. The adiponectin level was positively correlated with the mean carotid IMT measurements (*r *= 0.522, *P *= 0.004).

Four patients with TA (13.8%) had carotid atherosclerotic plaques. Among them, two had coronary artery involvement. There was a significant relationship between the occurrence of carotid atherosclerotic plaque and coronary artery involvement (*P *= 0.004).

## Discussion

Our study demonstrated decreased levels of circulating unacylated and acylated ghrelin in patients with TA. The ghrelin levels in patients with active disease were lower than those with inactive disease. Although the difference did not reach statistical significance, leptin levels in patients with active disease were increased. We also found that the leptin/ghrelin ratio was significantly higher in TA patients than in healthy controls. There was negative correlation between ghrelin levels and disease activity whereas the leptin/ghrelin ratio was positively correlated with disease activity. Furthermore, a reverse relationship was observed between leptin and ghrelin. The findings of decreased ghrelin and increased leptin in the patients who underwent treatment changes in the last month also supported the relationships of these hormones with active disease.

In the review of literature, we could find no study of the relationship of ghrelin and leptin with TA, and there was only one study investigating ghrelin levels in systemic vasculitis. In contrast to our results, this study showed that active anti-neutrophil cytoplasmic antibody (ANCA)-associated vasculitis is characterized by increased serum ghrelin and decreased serum leptin, both of which return to normal with successful therapy [22]. However, studies that have been performed in other forms of systemic vasculitis (including Henoch-Schönlein purpura and Behcet's disease) have shown increased levels of leptin in active disease periods [23-25]. Several studies have also reported increased leptin levels in systemic lupus erythematosus (SLE) [26-30] and rheumatoid arthritis (RA) patients [31-35], and some have demonstrated their correlation with disease activity [28,29,31-33,35]. There are a few reports on ghrelin that have revealed contradictory results in inflammatory rheumatic diseases. Increases in circulating ghrelin levels have been reported in ankylosing spondylitis (AS)[36] and RA. However, other studies have found significantly decreased ghrelin levels in patients with RA and in a rat adjuvant-induced arthritis model [37,38]. Decreased ghrelin levels have also been reported in patients with juvenile idiopathic arthritis. Furthermore, this last study has shown a significant relationship between ghrelin and disease activity [39].

All these studies confirmed the relationship of leptin and ghrelin with inflammation but it is unclear how the extent and severity of inflammation, the stage of disease progression, nutritional status and/or stress can impact this relationship. The other disease mechanisms may also directly or indirectly influence these hormones or the expression of their receptors in tissues. Thus, the discrepancies in the results of the studies may be explained by these factors as well as methodological differences.

Ghrelin has been shown to inhibit the leptin-induced cytokine expression in a dose-dependent manner, while leptin has upregulated ghrelin receptor-secretagogue receptor (GHS-R) expression on human T lymphocytes [9]. Leptin is known as a pure chemo-attractant and appears to have proangiogenic activity [40,41] whereas ghrelin inhibits angiogenesis [42,43]. These findings suggest the existence of a reciprocal regulatory network by which ghrelin and leptin control immune cell activation, inflammation and angiogenesis [9]. The present study also suggests a negative interaction between leptin and ghrelin.

In most of the studies, only total ghrelin levels were investigated and no attempts were made to differentiate between circulating acylated versus unacylated ghrelin levels [10]. In this study, we report a significant positive relation between the levels of these two forms of ghrelin in patients with TA and our results suggest that both of them are related to the inflammation.

Some studies have reported that low ghrelin levels are associated with type 2 diabetes, insulin resistance, and hypertension [44,45]. Therefore, we investigated the impact of comorbidities, including diabetes mellitus and hypertension, on the study results but we found no difference in acylated and unacylated ghrelin levels between TA patients who have these comorbidities and those who do not. Decreased ghrelin level under endogenously or exogenously induced hypercortisolism has been also reported previously [46], but we did not find any difference in unacylated and acylated ghrelin levels between patients using glucocorticoid treatment and those not using glucocorticoid treatment.

Although the level of adiponectin in patients with TA was not different from healthy controls, it was positively correlated with carotid IMT measurements, suggesting that there may be a relationship between adiponectin and disease activity. To our knowledge, there are no published data on adiponectin in TA or other systemic vasculitis.

Several studies have shown that BMI and waist/hip ratio positively correlate with leptin levels and negatively correlate with adiponectin and ghrelin levels in various disease groups and in healthy controls [47-51]. Therefore, we used a matching method to control for confounding effects due to anthropometric values. Meanwhile, our results were consistent with the previous studies that have shown these relationships.

The main limitation of our study is a lack of comparison of study variables between the pre-treatment and post-treatment periods in TA patients. However, there are several difficulties in performing a prospective study in TA, which is not a common disease [52,53]. There are problems in determining disease activity in patients with TA because a reliable marker is not yet available [54,55] and many patients with TA have persistent disease associated with chronic low-grade inflammation. ESR and CRP are the most frequently used tools in the assessment of disease activity but their sensitivity and specificity are quite low [54,55]. Serum IL-6, and regulated and normal T cell-expressed and secreted (RANTES) levels have been shown to be increased in active disease periods and correlated with the disease activity score in patients with TA. However, a larger sample size and additional studies are required to confirm these results. Other studies that have investigated several markers of endothelial injury, platelet activity, and thrombotic and fibrinolytic status have failed to show a significant relationship between these markers and disease activity [56,57]. In contrast, plasma endothelin-1 levels have been demonstrated to be positively correlated with ESR in patients with TA [58]. A recent study reported that plasma levels of pentraxin-3 (PTX3), an acute phase reactant, which is produced by vascular and immune cells in response to proinflammatory signals, has greater accuracy for distinguishing active from inactive disease than either ESR or CRP [59], but it needs to be studied in a larger group of patients with unknown disease activity. The value of these tests in follow up is still uncertain.

We previously demonstrated that there is a very good agreement between Kerr's criteria and DEI.Tak, and using Kerr's criteria instead of DEI.Tak increases the consistency with PGA [60]. The latter finding has suggested that the physician's decision could be influenced by the acute-phase reactants and imaging modalities. In the present study, we showed that radiological markers of disease activity are consistent with Kerr's criteria, DEI-Tak and PGA. Therefore, we suggest that the radiological markers may help to evaluate disease activity in patients with TA.

## Conclusions

This study demonstrated decreased levels of unacylated and acylated ghrelin in patients with TA. Patients with active disease had lower unacylated ghrelin levels than those with inactive disease and had lower acylated ghrelin levels than healthy controls. Ghrelin levels were negatively correlated with various parameters of disease activity. Furthermore, the leptin/ghrelin ratio was higher in TA patients than in controls. It was positively correlated with disease activity. There was a positive correlation between unacylated and acylated ghrelin and a negative correlation between leptin and ghrelin. No difference was determined in adiponectin levels between TA patients and controls.

The results of the study suggest that plasma unacylated and acylated ghrelin levels may be useful in monitoring disease activity and planning treatment strategies for patients with TA. The leptin/ghrelin ratio may also be used to help assess disease activity. Further studies are needed to validate the use of these laboratory tests in daily clinical practice.

## Abbreviations

ACR: American College of Rheumatology; ANCA: anti-neutrophil cytoplasmic antibody; AS: ankylosing spondylitis; BMI: body mass index; CRP: C- reactive protein; DEI.Tak: Disease Extent Index-Takayasu; DSA: digital subtraction angiography; ELISA: enzyme-linked immunosorbent assay; ESR: erythrocyte sedimentation rate; GH: growth hormone; GHS-R: ghrelin receptor-secretagogue receptor; IGF-1: insulin-like growth factor 1; IL: interleukin; IMT: intima-media thickness; MRA: magnetic resonance angiography; MRI: magnetic resonance imaging; PGA: physician's global assessment; PTX-3: pentraxin-3; RA: rheumatoid arthritis; RANTES: regulated and normal T cell-expressed and secreted; SLE: systemic lupus erythematosus; TA: Takayasu arteritis; TMB: 3,3',5,5'-tetramethylbenzidine;

TNF-α: tumor necrosis factor- alpha; US: ultrasonography; WBC: white blood cell.

## Competing interests

For all of authors there is no personal or financial relationship with other people or organizations that may influence the interpretation of data or presentation of information.

## Authors' contributions

HY and FO participated in the design of the study, acquisition, analysis and interpretation of data, and was involved in drafting the manuscript. VG participated in the design of the study, acquisition and interpretation of data. DK and DS participated in the design of the study, analysis and interpretation of data and was involved in drafting the manuscript. SA participated in the design of the study, acquisition, analysis and interpretation of data. GC, MB, and AG participated in the design of the study, and acquisition of data. YG participated in the design of the study and was involved in drafting the manuscript. IS participated in the design of the study, and interpretation of data. NA participated in the design of the study, and interpretation of data. All authors approved the final version of the manuscript for publication.
